# Compositional and structural analysis of Fukushima-derived particulates using high-resolution x-ray imaging and synchrotron characterisation techniques

**DOI:** 10.1038/s41598-020-58545-y

**Published:** 2020-01-31

**Authors:** Peter G. Martin, Christopher P. Jones, Silvia Cipiccia, Darren J. Batey, Keith R. Hallam, Yukihiko Satou, Ian Griffiths, Christoph Rau, David A. Richards, Keisuke Sueki, Tatsuya Ishii, Thomas B. Scott

**Affiliations:** 1 Interface Analysis Centre, School of Physics, University of Bristol, Bristol, BS8 1TL UK; 2 Diamond Light Source, Harwell Science and Innovation Campus, Didcot, Oxfordshire OX11 0DE UK; 3 Collaborative Laboratories for Advanced Decommissioning Science (CLADS), Japan Atomic Energy Agency (JAEA), Tomioka-Machi, Futaba-gun, Fukushima, 979-1151 Japan; 4 Department of Materials, University of Oxford, Oxford, OX1 3PH UK; 5 School of Geographical Sciences, University of Bristol, Bristol, BS8 1SS UK; 6 Faculty of Pure and Applied Sciences, University of Tsukuba, Tsukuba, Ibaraki 305-8571 Japan

**Keywords:** Environmental impact, Nuclear fusion and fission

## Abstract

Both the three-dimensional internal structure and elemental distribution of near-field radioactive fallout particulate material released during the March 2011 accident at the Fukushima Daiichi Nuclear Power Plant is analysed using combined high-resolution laboratory and synchrotron radiation x-ray techniques. Results from this study allow for the proposition of the likely formation mechanism of the particles, as well as the potential risks associated with their existence in the environment, and the likely implications for future planned reactor decommissioning. A suite of particles is analyzed from a locality 2 km from the north-western perimeter of the site – north of the primary contaminant plume in an area formerly attributed to being contaminated by fallout from reactor Unit 1. The particles are shown to exhibit significant structural similarities; being amorphous with a textured exterior, and containing inclusions of contrasting compositions, as well as an extensive internal void volume – bimodal in its size distribution. A heterogeneous distribution of the various elemental constituents is observed inside a representative particle, which also exhibited a Fukushima-derived radiocesium (134Cs, 135Cs and 137Cs) signature with negligible natural Cs. We consider the structure and composition of the particle to suggest it formed from materials associated with the reactor Unit 1 building explosion, with debris fragments embedded into the particles surface. Such a high void ratio, comparable to geological pumice, suggests such material formed during a rapid depressurisation and is potentially susceptible to fragmentation through attrition.

## Introduction

Following the 2011 magnitude 9.0 Great Tōhoku earthquake and subsequent tsunami off the eastern coast of Japan^1^, a considerable amount of radioactivity was released from the Fukushima Daiichi Nuclear Power Plant (FDNPP) into the global environment. Loss of coolant incidents (LOCI) associated with three of the sites nuclear reactors^2^ were responsible for the radioactive releases. Estimates place the total release from this International Nuclear Event Scale (INES) Level 7 event at between 340 PBq and 800 PBq^3^ – one tenth of that resulting from the Chernobyl accident 25 years earlier^4^.

Immediately after the accident, considerable research effort was invested into studying the radiocesium strongly adsorbed to phyllosilicate minerals in sediments^5^. However, when Adachi *et al*.^6^ first reported the isolation and analysis of infrequent, but considerably more active, particles (averaging 2 μm in diameter) from material obtained 170 km southwest of the FDNPP, attention was diverted to alternative forms of Fukushima-derived contamination. Progressing the analysis of Adachi *et al*., who employed scanning electron microscopy (SEM) combined with energy dispersive spectroscopy (EDS), work by Abe *et al*. examined the internal structure of some of these micron-scale particles using synchrotron-radiation (SR) x-ray fluorescence (XRF)^7^. The subsequent discovery of the more wide-spread dispersion of such micron-scale fallout material prompted further investigations on the structure and composition of such spherical material using an expanding suite of advanced analytical techniques^8–11^, the most common being transmission electron microscopy (TEM). By comparing the measured radiocesium activity ratios (^134^Cs/^137^Cs) of this material with reactor-core inventory modelling^12^, these micron-scale “Cs-balls” were attributed to the emission from reactor Unit 2^13,14^.

In contrast, material derived from closer to the plant, located more northerly than the main plume produced by Unit^2^, has received scant scientific attention. Termed “Type B” by Satou (2016)^13^, this similarly amorphous material is composed predominantly of Si glass and is sub-millimeter in maximum dimension. Its ^134^Cs/^137^Cs activity ratio (<1.0) invokes a source attributable to reactor Unit 1 and a release on the 12th March 2011 (Unit 3 is excluded because of the prevailing wind conditions at the time^15^). Preliminary SR-µ-XRF by Ono *et al*.^14^ on sub-regions of Unit 1 particulates indicated a high degree of compositional heterogeneity, with all of the elements encountered within these samples attributable to either reactor fuel/components, or fission products. While the heterogeneous distribution of species was shown by Ono *et al*., only a two-dimensional visualisation was produced, with no insight into the materials internal structure. Such structural information is an important component in defining both the material provenance and its environmental impact/legacy, as well as elucidating material properties applicable to imminent decommissioning activities. Recent mass-spectrometry analysis on dense surface-bound inclusions within a suspected Unit 1 particulate^16^, identified the presence of micron-scale U fragments, that upon characterisation were definitively shown to have been spent fuel fragments derived from reactor Unit 1 at the FDNPP.

The characterization of radioactive particulate from other release scenarios has been reported extensively within the scientific literature, with the earliest works focusing on fallout material originating from atmospheric nuclear weapons testing^17^. A comprehensive review of the various methods available to study such environmentally sourced material was undertaken by Salbu and Krekling (1998)^18^. This was succeeded by the IAEA TECDOC 1663^19^ (and contributing works therein).

While non-destructive x-ray tomography (XRT) had been a mainstream technique within materials analysis for a number of years^20^, it was first applied to nuclear-derived particulates by Salbu *et al*.^21^, where it was used to examine environmentally-sourced spent-fuel samples released following the 1986 Chernobyl accident. Using synchrotron radiation to provide the incident x-ray beam, a 0.6 μm XRT spatial resolution was computed. This work also featured the combined application of SR-μ-XRF and SR-μ-x-ray absorption spectroscopy (XAS) to examine the composition and oxidation state chemistry of the sample.

Our study follows the work on similar Chernobyl-derived material by exploring the source and formational environment of FDNPP-sourced particulate. We consider the long-term environmental impacts of the disaster by illustrating the material characteristics and the potential susceptibility to breakdown, mobility and contamination risk. We further evaluate the implications for the future decommissioning of the material from within the reactor based on its properties. In this work, laboratory based XRT analysis is applied to a suite of reactor Unit 1 derived particulates collected from close to the perimeter of the plant to observe (for the first time) the materials internal form. Subsequent SR-µ-XRT combined with SR-µ-XRF is then applied to a representative fallout particle to examine the compositional variation. This work follows the previous study by the authors^16^, and their first identification of U composition particulate inclusion contained within one of the sub-mm particles, through which it was possible to attribute the larger particle to a specific on-site reactor source. In this follow-on study, an enhanced analysis of the structure of the particulates and their internal elemental distribution is undertaken, through which it is possible to understand the likely conditions of formation, their environmental legacy and the resulting decommissioning challenge – beyond the indicative results from a single U-containing reactor-derived particle.

## Results

### Energy dispersive spectroscopy and gamma-ray spectroscopy

**Figure 1:**
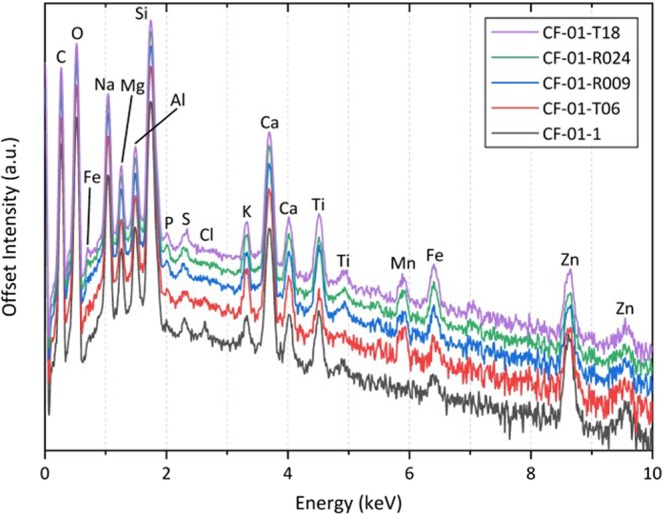
EDS spectra obtained from the five reactor Unit 1 particles; CF-01-T18, CF-01-R024, CF-01-R009, CF-01-T06, and CF-01-1.

The results of whole-particle EDS analysis on each of the five particle samples (CF-01-R024, CF-01-R009, CF-01-T18, CF-01-T06, and CF-01-1) are shown in Fig. 1. As observed in earlier works 13,14, the average constituents comprising each particle are; Si (40 wt%), C (17 wt%), O (14 wt%), Na (9 wt%), and Ca (8 wt%). Additional elements consistent with these earlier studies include; Mg, Al, K, S, P, Mn, Fe and Zn (ranging from 6 – 1 wt%). The work by Ono *et al*.^14^ also detected Ti, Pb and Cr in some particles, although only Ti was observed (albeit within all particulates) in this instance. The suite of elements observed here is similar to that obtained for smaller, spherical material said to have been derived from Unit 2^6,7^. The one fundamental difference between the smaller (spherical) Unit 2 material and these larger reactor Unit 1 particles, however, is the absence of Cs from the EDS spectra (where absolute detection limits are ~ 0.1 wt%). For the most-radioactive particle analyzed, CF-01-1, the decay-corrected (to the incident date of the 11th March 2011) ^134^Cs and ^137^Cs activities are 18,000 Bq and 19,300 Bq respectively, with a ^134^Cs/^137^Cs activity ratio of 0.93 – confirming its provenance from FDNPP, and to reactor Unit 1^12^. At the time of this study, ^134^Cs and ^137^Cs activities are 1,800 Bq and 16,800 Bq respectively, which translates to a combined ^134^Cs and ^137^Cs abundance of approximately 39 pmol.

Even for this most radioactive particle, the concentration of radiocesium in the material is more than four orders of magnitude less per unit volume than the smaller Unit 2 material 13,14. Recent isotopic analysis using secondary ion mass spectrometry (SIMS) of (albeit smaller) particulate matter by Imoto *et al*.^22^, determined a ^137^Cs/^133^Cs atomic ratio ≈1 – implying comparable abundances of both stable and fissionogenic Cs. While naturally-occurring (^133^Cs) is widely-encountered within the environment (within soils, plants and crops)^23,24^ (as well as being a trace yield fission product^25^), a number of works examining the composition of the micron-scale Cs-bearing microparticles or “Cs-balls” (attributed to reactor Unit 2), determined all of the Cs (irrespective of isotope) to be associated entirely with the very-outermost regions of the smaller particles^9,11^. This notable surface enrichment (<0.5 μm) of these spherical samples reduces to concentrations below the limit of detection towards the center (transmission electron microscopy – TEM, coupled with EDS).

### Internal particle morphology

**Figure 2:**
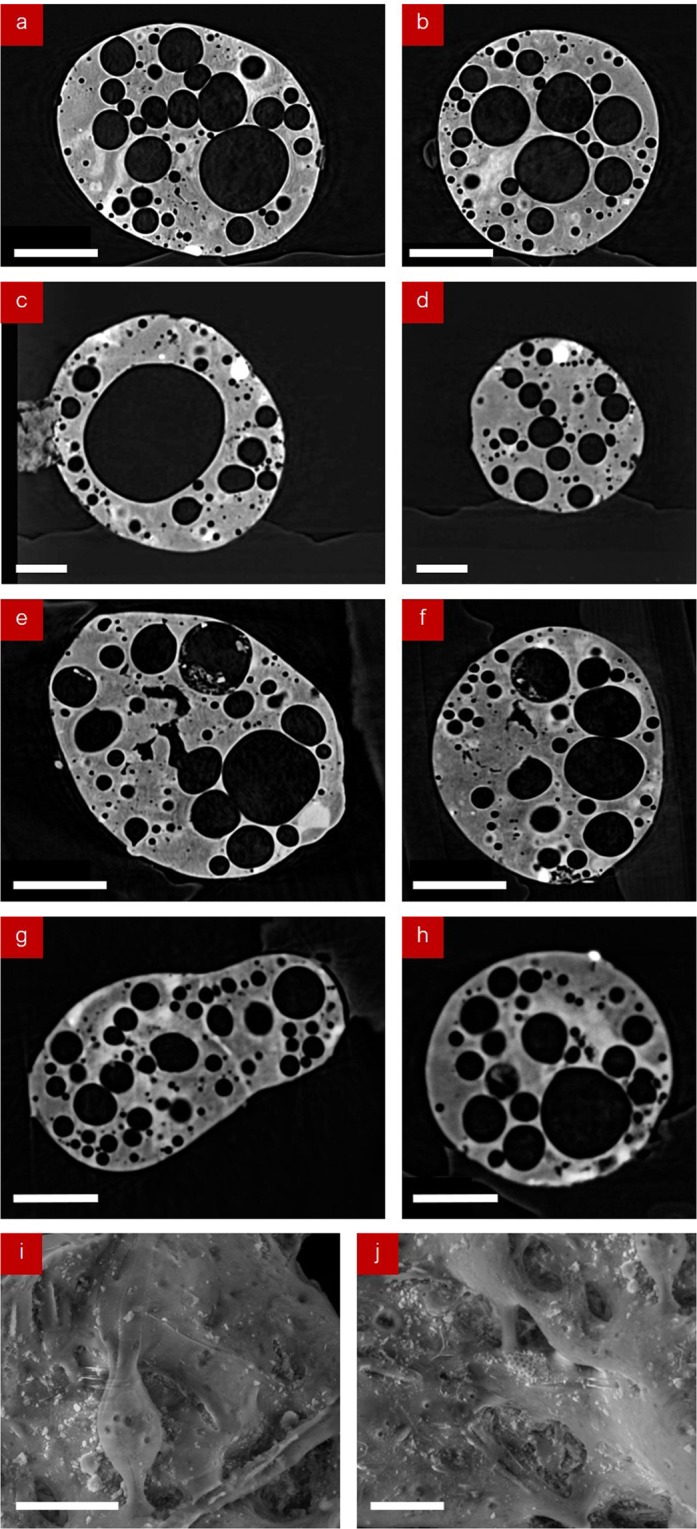
Orthogonal tomographic (absorption contrast) sections through the central XY (horizontal) and YZ (vertical) planes of the particles; (**a**,**b**) CF-01-R024, (**c**,**d**) CF-01-R009, (**e**,**f**) CF-01-T18, and (**g**,**h**) CF-01-T06. (scale bars: a – h = 100 μm, i and j = 10 μm).

Orthogonal laboratory x-ray tomography (absorption) sections of particles CF-01-R024, CF-01-R009, CF-01-T18, and CF-01-T06 taken along the median horizontal and vertical planes are shown in Fig. 2(a–h), respectively. For all four particles, each section reveals the existence of a significant internal volume of spherical voids. Observed within each radiograph is the highly spherical nature of these voids, the largest of which (Fig. 2(c)) is approximately 300 μm in diameter. Alongside these voids, through the variation in x-ray absorption contrast, the residual artefacts of a fibrous structure are observed to exist within the (solid) bulk material of each particle. This structure is illustrated in the SEM images (due to high x-ray transmission for such Si-based fibres) of the surface of sample CF-01-R024, shown in Fig. 2I,j.

**Figure 3:**
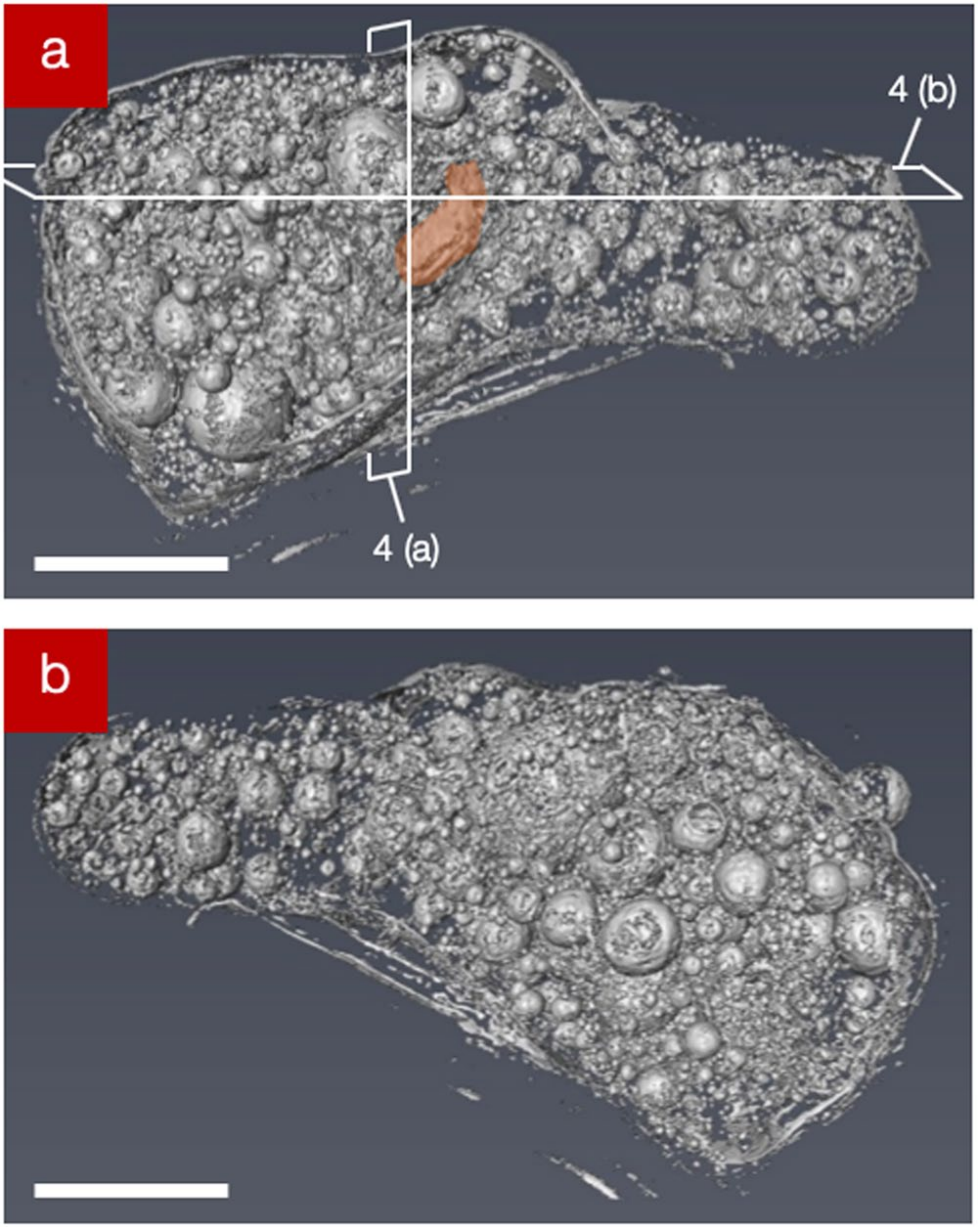
(**a**) SR-μ-XRT surface rendering of the “front” face of the fallout particle, with the location of Fe-rich inclusion highlighted with the lines of section (Fig. 4). (**b**) SR-μ-XRT surface rendering of the “back” surface of the particle. (scale bars = 100 μm).

Iso-surface renderings of both the “front” and “back” of the fifth particle (CF-01-1) analysed more-extensively using SR-µ-XRT (following initial characterisation using laboratory XRT) are shown in Fig. 3(a,b), respectively. This particle was selected for enhanced synchrotron examination due to the greatest levels of radioactivity it contained. As exhibited by the aforementioned four particles, these renderings show a similarly highly complex and heterogeneous internal structure, with many near-spherical voids spanning a wide range of sizes – the largest of which measures 80 µm in diameter. In contrast, the smallest void observed in this particle occurs with a diameter of less than 0.8 µm (limit of image reconstruction resolution). The particle is similarly shown to possess a foam-like internal structure, with regions of the exterior surface observed to be both highly smoothed/glassy in form but also contrastingly highly textured across neighboring areas. For this particle (CF-01-1) we calculate that 24% of the internal volume to be comprised by void (free) space. This value is consistent with the porosity values observed in the other four particles; CF-01-R024 (28%), CF-01-R009 (31%), CF-01-T18 (24%), and CF-01-T06 (26%).

**Figure 4:**
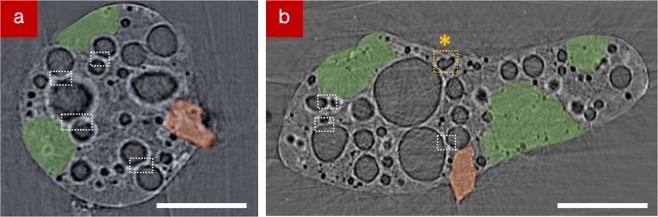
(**a**,**b**) SR-µ-XRT sections, obtained vertically and horizontally, through the 3-dimensional particle reconstruction (as identified by the lines of section in Fig. 3(a)), with the location of the Fe-rich particle (orange) and Ca-rich regions of low porosity (green) identified (scale bars = 100 µm). The locations where voids connect are also shown (white boxes), with the location at which two bubbles have likely fused additionally highlighted (yellow box and *).

In addition to this considerable porosity, a large solid fragment (subsequently identified as Fe-based via SR-μ-XRF and determined as being comparable to a steel using EDS analysis - Table S1) is observed embedded into the outer surface of the CF-01-1 sample. The location of this angular particle is highlighted in Fig. 3(a) and is further detailed in the corresponding tomographic slices through the sample in Fig. 4(a,b). In these orthogonal sections, taken both vertically and horizontally through the sample, the fragment (highlighted orange in Fig. 4(a,b)), is observed to represent the only prominent extrusion outside of the otherwise well-rounded/featureless particle surface.

**Figure 5:**
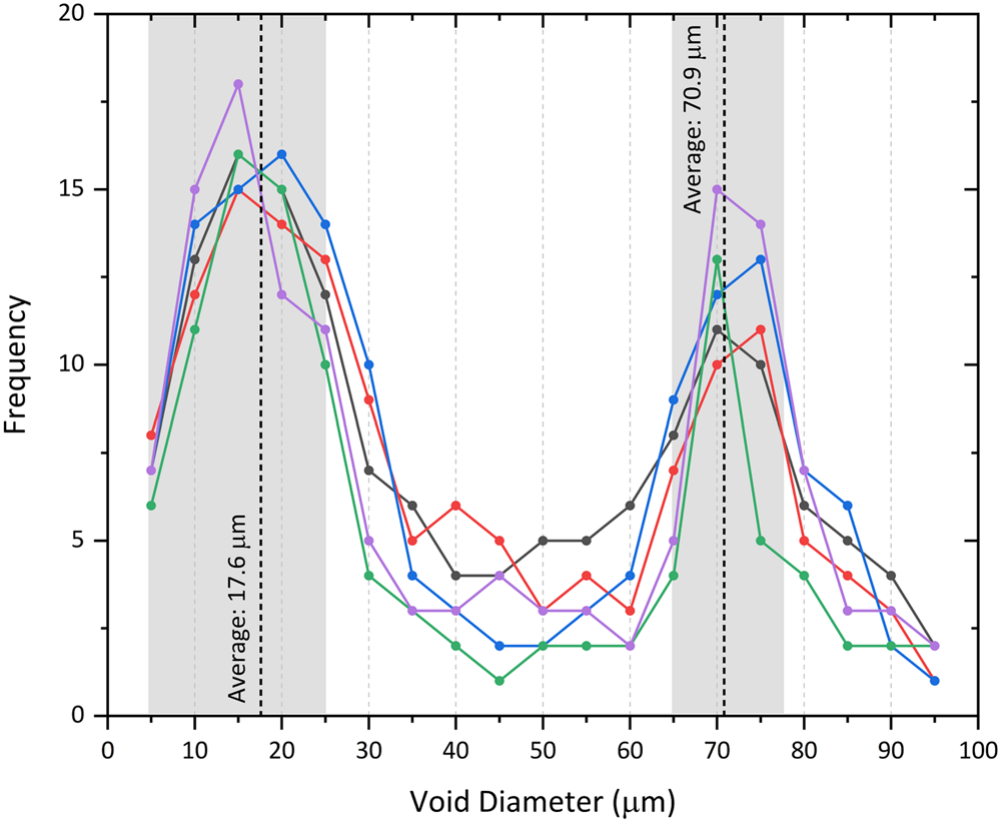
Size distribution (void diameter) of the spherical pores in each of the five particulate samples.

In addition to the highly spherical nature of the voids within all five of the particles analysed, (Figs. 2–4) we further observe a bi-modal size distribution of these structures. This bi-modal distribution of the void diameter for all five isolated particles is illustrated graphically in Fig. 5. With mean diameters of the larger voids at 70 µm, the smaller voids occur with diameters between 5 μm and 25 μm (alongside a very minor component of such voids existing as small as 2 µm). As shown in the tomographic sections of Figs. 2 and 4(a,b), the smaller (<25 μm) voids are observed to exhibit a greater concentration around the circumference of the particles than the larger pores. Together, both the larger and smaller voids are distributed throughout the particle, except for several sub-100 μm regions where very few (or none) of either size voids exist (shaded green in Fig. 4(a,b)).

While the fragment extruding from the surface (shaded orange in Figs. 3(a), 4(a,b)) is shown by EDS analysis to be compositionally compatible with a steel (Table S1), the larger regions exhibiting lower porosity (shaded green in Fig. 4(a,b)) are conversely Ca-rich. Through additional EDS x-ray analysis we determine these regions to be compositionally analogous to a cement, with the average EDS results of these regions shown in Table S2, alongside a reference composition for a typical Portland-type cement^26,27^.

### Bulk composition

**Figure 6:**
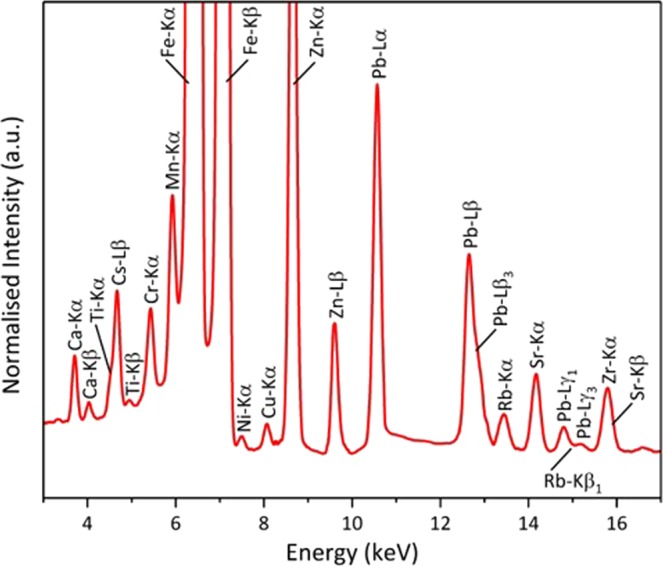
SR-µ-XRF spectrum obtained from the entire particle volume, with the contributing characteristic fluorescence emission peaks identified.

A SR-μ-XRF spectrum obtained from the entire volume of the representative CF-01-1 particle is shown in Fig. 6. While the characteristic x-ray emissions at less than 3 keV (principally Na, Mg, Al and Si) are below energies attainable on the beamline, a composition similar to that obtained in prior SR analysis work on Unit 1-type material is attained^14^ – with a considerable Fe and Pb content. Unlike the results attained via EDS analysis of the material, however, the x-ray emission peaks from SR-μ-XRF detail the presence of Cr, Ni, Cu, Pb, Rb, Sr, Zr, Ba and Cs in the sample (owing to the techniques’ enhanced limits of detection). The study by Ono *et al*.^14^, identified several other elements that are not identified during this analysis due to either their potential absence (Nb, Mo, U) or more-fundamentally their characteristic x-ray fluorescence emissions occurring at energies beyond those achievable on the beamline, i.e. 19 keV (Ag, Cd, Sn, In, Pd, Sb, Te and Ba). Therefore, although a comparable size, exterior surface form and ^137^Cs activity (Table S3) are evident between the particles of this study, there exists a degree of compositional variability between similarly sourced particulates. Such characteristics are not unexpected, as despite being of similar form, an analogous “inter-group” compositional variability was observed for particulate material released from the Chernobyl accident^28,29^.

### Species distribution

**Figure 7:**
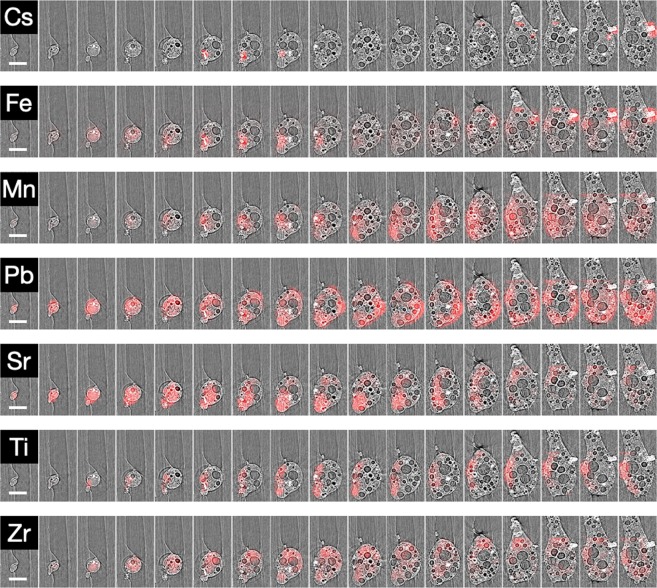
SR-µ-XRT sections through the lower portion of the particle, overlain with SR-µ-XRF data. The location of species is determined following a background spectrum (Kapton) subtraction from the signal obtained at every other stage position. (scale bar = 100 μm).

Applying the SR-μ-XRF results to longitudinal slices through the three-dimensional SR-μ-XRT iso-surface rendering of the particle (Fig. 3(a,b)) yields the three-dimensional compositional results displayed in Fig. 7. From these reconstructions, considerable compositional heterogeneity is exhibited.

Of note is the distribution of Cs. During previous studies (on Unit 2 particulate), Cs was observed to be spatially heterogeneous and concentrated within the outer “rind”, or skin, of the Si-based glass matrix^7–9,11^. As is shown in Fig. 7, Cs in this particulate is observed to be similarly heterogeneous – and exists in only a few highly concentrated locations. These locations are associated primarily with that of the Fe-rich regions as well as around the circumferences of several of the smaller-sized (and perimeter concentrated) voids. A numerical (per pixel) quantification of this Fe and Cs correlation (Pearson correlation coefficient) of 0.82 strongly illustrates the co-existence of Cs with Fe within the majority of the 10 μm × 10 μm × 10 μm cubic mathematical voxels.

In addition to Cs and Fe, similarly spatially discrete compositional regions are observed for Sr, Ti and Zr. In contrast, both Mn and Pb are seen to exhibit a spatially extensive and diffuse elemental distribution. For these elements, however, such distributions (observed here for the first time in three-dimensions) contrast with those of material sourced from Unit 2 – but in agreement with the results obtained previously on a small sub-region of a similar Unit 1-attributed particle by Ono *et al*., (2017) ^14^.

The magnetic sector-secondary ion mass spectrometry (MS-SIMS) analysis of a Cs-rich region of the sample is shown in Fig. S3, wherein only complementary mass peaks at 134 amu, 135 amu, 136 amu and 137 amu, are observed. Alongside highlighting the absence of a naturally occurring (non-fissionogenic) ^133^Cs component, the lack of such a ^133^Cs contribution is consistent with the isotope’s very low fission product yield (with 5 orders of magnitude less produced than ^137^Cs). This deficit of ^133^Cs contrasts with the results of Imoto *et al*.^22^, where comparable abundances of both ^133^Cs and ^137^Cs were encountered (albeit for the smaller Unit 2-derived “Cs-ball” particulate).

## Discussion

Having formerly identified Si as constituting the bulk of the particle (by EDS analysis), there exists only a limited number of potential sources of such Si within the confines of reactor Unit 1 – with this material occurring solely as Si-based thermal insulation at a limited number of areas around the reactor unit (personal communication with TEPCO engineers); (i) enclosing the pipework of the heat-exchanger network, (ii) in the roof-space of the reactor building, or (iii) surrounding the Primary Containment Vessel (PCV), which encompasses the main reactor pressure vessel (RPV) – itself containing the reactors fuel assemblies. The most extensive volume of fibrous Si-based thermal material was situated around the PCV, occurring next to the structural cement of the reactors underlying foundation pedestal and the extensive volume of steel that formed the RPV 30. The specific type of thermal insulation material used at the facility is the highly fibrous glass-fibre material.

Such types of fibrous material are produced through melting and extrusion of either silicate rocks (basaltic species) or recycled glass, with the former possessing a typical mean diameter of 4 μm – 5 μm and length >200 μm^30^. This provenance, combined with the similarity of the particulates internal structures to that observed for naturally-occurring volcanic pumice^31^, allows for a melting mechanism to be invoked to account for this apparent bi-modal void distribution. A schematic representation of which is shown in the illustrations of Fig. S4.

As has been widely-reported – at the time of the accident, a large volume of gas existed within the environment surrounding the reactor as a result of venting of the Unit 1 primary containment^32,33^. This gas was composed of fission product/noble gas species, alongside a large quantity (approximately 130 kg) of hydrogen^34^, which was produced as a result of the autocatalytic H_2_ – O breakdown of the reactors coolant water in the presence of Zircaloy-4™ cladding at the elevated temperatures experienced during the LOCI.

The smaller voids (with average diameters between 5 μm and 20 μm, but also occurring as small as 2 μm) are ascribed to result from the incorporation of gases produced under the accident conditions – forming first, before being quickly solidified during quench-like cooling. SR-µ-XRF identifies the location of Cs and Sr fission products as corresponding to the higher concentrations around the perimeters of such smaller pores, and not associated however, with the larger voids. (N.B. Cs is also observed to correlate with the locations of Fe-based particulate in the sample). Owing to this Cs distribution, the significant fission product and hydrogen overpressure surrounding the reactor unit is believed to have resulted in the formation of these voids, which are of greater abundance around the perimeter of the particle.

Upon heating and partial melting of the source silicate material during the accident, volatile species originally contained in the once fibrous material were exsolved to produce a considerable gaseous volume, therefore resulting in the larger diameter internal voids evident within the particulate. The highly spherical nature of these large bubbles suggests that they were not produced by gases flowing into the material, rather growing *in-situ*, via exsolution of dissolved gases and volatiles as the molten silica was rapidly depressurised and cooled adiabatically in the time subsequent to the reactor building’s hydrogen explosion. Resulting from their size, these larger, more centrally located, bubbles represent a reduced rate of cooling seen in the particle interiors whereby smaller gas bubbles had time to either grow or coalesce to form the greater diameter voids observed. This same phenomenon is witnessed within volcanic pumice (albeit on larger scale lengths) wherein volcanic bombs, which are molten eject, exhibit a frozen rim/rind containing smaller vesicles with much larger vesicles entrapped within the core of the material resulting from a reduced rate of cooling.

The locations at which interactions occur between the voids are highlighted additionally within Fig. 4(a,b). Here, the larger voids are observed to deform around the smaller voids – resulting in a slight degree of deformation as well as highlighting the relative order of formation, with the smaller voids emplaced and solidified initially and the subsequent larger voids distorting around the bubbles located within the solidified rim. The amalgamation of bubbles (* in Fig. 4(b)) may also contribute to the formation of larger diameter voids. To investigate such potential compositional variance associated with these bi-modal size voids, future work will apply techniques such as focused ion beam (FIB) ablation coupled with residual gas analysis (RGA) or SIMS to etch the particles surface (and into the voids) to liberate and isotopically analyze any species that may have condensed onto the internal surfaces of the voids.

A specific element of interest observed at low concentrations (identified in the EDS analysis shown in Fig. 1) is Cl. As is shown in Table S4, a bulk EDS analysis of virgin precursor (glass) fibrous material, Cl is not observed at detectable levels (<0.1 wt%). Consequently, such occurrence within the particulate would suggest it was incorporated into the particulate’s matrix, subsequently, during formation. One of the primary sources of Cl- within the Unit 1 reactor environment at the time of release was seawater, injected during emergency core cooling. This large input represents the most likely source of free Cl− (and some Na+) into the particulate material, having been diffused, absorbed or volatilized into the silicate precursor, that existed at elevated temperatures – before being subsequently fragmented during the violent H2 gas ignition and explosion.

The explosion of the contained hydrogen volume was hence sufficiently violent to fragment the once-molten and once-fibrous Si insulation material, as well as also being incorporated into it. Regions within the sample where no pore volume exists result from the inclusion of pieces of structural cement – incorporated into the once-molten sample. Such Ca-rich regions occur alongside the small protruding steel composition fragment. A difference in the hardness values that exists between the harder Fe (steel) and softer cement^35^, most likely accounts for the variation in surface form – with the cement having been abraded as a result of the explosion (while the steel conversely remained protruding from the surface).

Combined, these characteristics permit for the environmental affinity (over a range of timescales) of this and other such environmentally dispersed material, to be assessed. Like similar geologically-derived samples, the “pumice-like” form of this particulate would suggest that it could be readily weathered under ground surface conditions^31^. However, the 3.7 years that this material existed within the Fukushima environment (between March 2011 and sampling in November 2014) does, however, imply a level of tolerance to surface processes. The extent to which any weathering/erosion may have occurred prior to the materials isolation from the environment represents a significant unknown. However, the form of all of the particulates, with no concave – only convex features, suggests that both physical attritional and chemical weather have combined, not extensively eroded through the Si-based material into the void(s), therefore indicating a hard material strength.

While a component of the radiocesium is associated with the perimeter of the smaller-diameter pores (<20 μm), the SR-μ-XRF measurements identify the bulk of this Cs to be associated with the Fe-based inclusions. As presented in the study on Chernobyl-derived particulate by Al Rayyes *et al*.^36^, a strong Fe-Cs affinity (with the formation of ferrites) was observed, resulting in the solubility of Cs (when attached to insoluble reactor structure-sourced Fe particulate) within the environment being greatly reduced. This Fe surface bonding of Cs will therefore lead to the production of insoluble Fe-Cs particles rather than a readily-soluble form of environmental Cs^37^. The analogous “vitrification” of such material within a glassy matrix will additionally serve to reduce the potential leaching of Cs or any other associated fission product species^38^, suggesting that these particles are potentially less susceptible to attritional environmental weathering than previously assumed – although containing a reactor-sourced uranium component^16^. During future decommissioning activities, the existence of such material could represent a significant difficulty or hazard for removal, with the occurrence of a greater void volume and fragment inclusions yielding a material for removal that is more brittle and readily friable. Further work is therefore needed to assess the influences of these features on the materials properties.

## Materials and Methods

### Sample collection and preparation

Contaminated bulk sediment and dust material was collected in November 2014 from 37.4379° N, 141.0222° E, a site located 2 km north-west of the center of the plant within the highly contaminated “restricted zone”. This material exhibited high count-rates at the site of sampling. Similar source material was previously analysed within the work of Ono *et al*.^14^. To isolate the radioactive particles from the surrounding matrix material, a multi-stage autoradiography and division process was employed^39^. An exposure time of only 5 minutes on the imaging plate (IP, GE Measurement and Control CRX25P digital radiography scanner) ensured only the most radioactive particles were identified and extracted using a manual micro-manipulator (AP-xy-01, Micro Support Corporation). The initial autoradiography image of the bulk sample, with darkening associated with the most-radioactive particle (CF-01-1), is shown in Fig. S1. Following this, the particle was then placed onto a piece of adhesive Kapton film (DuPont Ltd.) for initial scanning electron microscopy (SEM)/energy dispersive x-ray spectroscopy (EDS) analysis and gamma-ray spectrometry measurements. Subsequently, each sub-mm particle was then encased within a double layer of Kapton film.

### Gamma-ray spectroscopy

To derive quantitative spectroscopic results of the gamma-ray emissions, each particle was analysed by placing it within a High Purity Ge (HPGe) detector (GC4018) from CANBERRA with associated multi-channel analyser. A calibration of the detectors efficiency was made through the use of a standard mixed gamma source provided by Japan Radioisotope Association. The counting time for each particle was varied in each instance to record a total of 10,000 counts of ^134^Cs (net count).

### Scanning electron microscopy (SEM) and energy dispersive spectroscopy (EDS)

Examination of the surface texture of each particle alongside derivation of bulk compositional spectra from the entire particle was conducted using a Zeiss SIGMA™ Variable Pressure (VP) Scanning Electron Microscope (SEM) equipped with an Octane Plus™ Si-drift characteristic x-ray energy dispersive spectroscopy (EDS) detector from EDAX (AMETEK Ltd.). All compositional characterisation (and imaging) was performed using a consistent 25 kV accelerating voltage, 120 µm aperture and 9 mm working distance. Each EDS spectrum was acquired for a period of 200 seconds (live time) to yield appropriate peak intensity. Control of the detector and processing of the results was performed using the associated EDAX TEAM™ software.

### Laboratory X-ray tomography

Resulting from the limited amount of synchrotron analysis time available, a pre-screening of the particulates was undertaken using complementary laboratory XRT to identify regions of interest for subsequent synchrotron x-ray examination. Although the laboratory instrument was capable of obtaining finer absorption/contrast tomography voxel resolutions (than synchrotron methods) of 50 nm, it was not possible to derive true compositional information (from x-ray fluorescence) using the laboratory system, unlike the coincident XRF and XRT data (albeit at reduced resolutions) obtainable using synchrotron radiation.

Each particle sample enclosed within the x-ray transparent Kapton film was mounted onto a stainless-steel support pin, which itself was attached to a magnetic base to ensure secure placement on the instruments multi-axis translation stage (Fig. S2). A Zeiss Xradia 520 Versa µXRT was used to obtain 3D tomographic data of the samples, which operated at 80 kV with no additional filtering. Collection was obtained using either the 20× or 40× optical magnification, depending on the sample size, with the generated image collected on an ANDOR low light camera.

Statistical analysis and quantification of the total porosity within the particles was performed on the.tiff slices generated from each of the tomographic reconstructions. The opensource ImageJ software suite and image processing plug-in^40^, was used to determine the proportion of each slice that was composed of free space (black), in contrast to that composed of solid particle (grey).

### Synchrotron radiation X-ray analysis

SR x-ray analysis of the representative particle (CF-01-1) was performed on the I13-1 (coherence branch) beamline at the Diamond Light Source (Harwell, UK) employing the same mounting setup as for previously detailed laboratory XRT analysis. With 250 m between the insertion device (undulator) and experimental end-station, the beamline benefits from highly-coherent x-rays with an energy range of 4 keV – 23 keV (19 keV used in this study). During the experiment, a coherent flux of 109 photons/second was attained, with a lateral coherence length of 400 µm (tuned through front-end slits). As is typical for the Diamond Light Source facility, a brilliance of 4 × 10^19^ photons/second/mm^2^/mrad^2^/0.1%BW (at 9.7 keV) was experienced, coupled to horizontal and vertical electron beam emittances of 2.6 × 10^−9^ m and 8.81 × 10^−12^ m, respectively.

Separate scans of the particle were performed during both SR-µ-XRT and SR-µ-XRF characterisation. For SR-µ-XRT analysis, the entire sample was illuminated with a 19 keV collimated x-ray beam (directly from the undulator, focused only using standard beamline “beam slits”) while being rotated through 180° in 1800 incremental steps to acquire three-dimensional sample projections using an optical microscope objective coupled to a 26 µm GGG:Eu scintillator providing a ×20 image magnification. This experimental setup resulted in a pixel size (resolution) of 0.45 µm. For the SR-µ-XRF, Kirkpatrick-Baez (KB) mirrors were used to focus the 19 keV beam onto the sample. A 10 µm pinhole located upstream of the sample was additionally used to remove any undesired “wings” from the focal spot. The sample was translated through the incident beam in a raster (snake) pattern at 10 µm steps and rotated through 180° in 40 incremental steps to acquire three-dimensional sample information. As a result, “edge effects” from the 10 µm aperture, positioned upstream of the sample, had to be considered. The effective resolution of this method is a 10 µm pixel size.

To obtain SR-µ-XRF data, a single-element Vortex fluorescence detector was used, positioned perpendicular to the sample and incident beam. An x-ray camera placed downstream of the sample was used to acquire data on the amount of sample self-absorption that occurred at each stage position for SR-µ-XRT analysis. From the series of projections, the three-dimensional volume for selected elements was reconstructed using the ordered-subset penalised maximum likelihood algorithm, with weighted linear and quadratic penalty algorithms in the TomoPy framework^41^. An iterative algorithm to correct for the degree of sample self-absorption that occurred was also employed for the tomography results (publication in preparation). The reconstructed three-dimensional images (absorption and fluorescence) were rendered using the Avizo software package^42^. To plot the spatial distribution of elements derived through XRF mapping, the normalised background spectra acquired at a stage position comprising only the Kapton film was subtracted from each of the subsequent sample-containing stage positions.

Statistical analysis and quantification of the coincident distribution of Fe and Cs within the particulate (as determined through SR-μ-XRF mapping) was performed through a spectral intensity (contribution) analysis of the gridded/pixelated sample volume. For each of the 10 μm × 10 μm × 10 μm pixels, the total contribution from Fe and Cs signals was deduced by determining the total counts that occurred within both primary (energy) emission windows. Using these values, a Pearson correlation coefficient (r) was determined to produce a numerical value representative of the linear correlation between the two variables.

To determine the presence/absence of elements where their differing characteristic emission lines overlapped (e.g. Zr-Kα and Sr-Kβ, and Ti-Kα and Cs-Lβ), additional processing of the XRF signal was undertaken. This was achieved through first considerably refining the energy window(s) within the spectrum attributable to the elements of interest (albeit at a slight detriment to the total signal intensity) to include only specific 10 eV wide regions of the spectrum. Alongside this narrowing of the permissible energy (channel) widths, the known intensity relationships that exist between the isolated peaks of the Sr-Kα and the Ti-Kβ emissions and their overlapping Sr-Kβ and Ti-Kα peaks (20% and 15% respectively^43^) were used to remove their contributions from the further additionally contributions of the Cs-Lβ (4.62 keV) and the Zr-Kα (15.72 keV) peaks.

The total time required to perform the complete 3D SR-μ-XRF compositional analysis of the particle on the I13-1 (coherence) beamline was 4 hours – with the proceeding tomographic rendering of the sample requiring less than 1 hour. While not as pronounced as the rate increase associated with the use of SR over laboratory x-ray sources (owing to the considerably greater photon fluxes), the analysis time between beamlines further varies dependent on differences in the specific source fluence and optical setups.

## Supplementary information

Supplementary Information.
