# Supplementary material for: Compositional and structural analysis of Fukushima-derived particulates using high-resolution x-ray imaging and synchrotron characterisation techniques
Source: Sci Rep. 2020 Jan 31;10:1636. doi: 10.1038/s41598-020-58545-y (PMC6994464; doi:10.1038/s41598-020-58545-y)
Supplement: Supplementary file 1 — Supplementary Information. [file 41598_2020_58545_MOESM1_ESM.docx]

Supporting Information for:

Compositional and structural analysis of Fukushima-derived particulates using high-resolution x-ray imaging and synchrotron characterisation techniques.

Peter G. Martin ^1^ *, Christopher P. Jones ^1^, Silvia Cipiccia ^2^, Darren J. Batey ^2^,

Keith R. Hallam ^1^, Yukihiko Satou ^3^, Ian Griffiths ^4^, Christoph Rau ^2^,

David A. Richards ^5^, Keisuke Sueki ^6^, Tatsuya Ishii ^6^, Thomas B. Scott ^1^.

^1^ Interface Analysis Centre, School of Physics, University of Bristol, Bristol, BS8 1TL, UK

^2^ Diamond Light Source, Harwell Science and Innovation Campus, Didcot, Oxfordshire, OX11 0DE, UK.

^3^ Collaborative Laboratories for Advanced Decommissioning Science (CLADS), Japan Atomic Energy Agency (JAEA), Tomioka-Machi, Futaba-gun, Fukushima 979-1151, Japan.

^4^ Department of Materials, University of Oxford, Oxford, OX1 3PH, UK.

^5^ School of Geographical Sciences, University of Bristol, Bristol, BS8 1SS, UK.

^6^ Faculty of Pure and Applied Sciences, University of Tsukuba, Ibaraki 305-8571, Japan.

** corresponding author*

*Email: peter.martin@bristol.ac.uk Telephone: +44 (0) 117 33 17684*


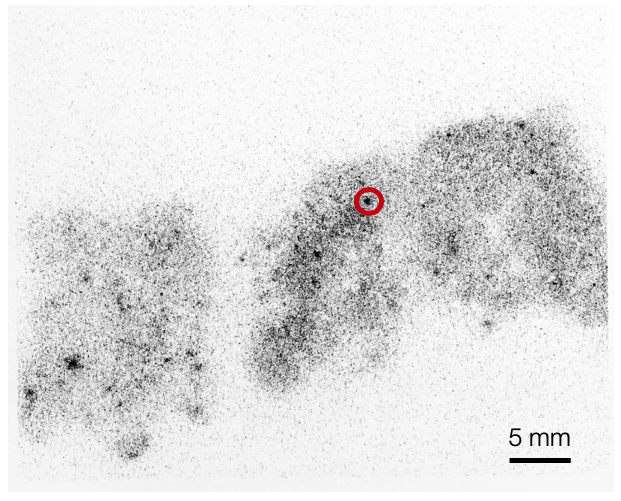


***Figure S1.*** *Autoradiography image of the bulk sediment sample, the location of a highly active particle (CF-01-1) subsequently removed is circled.*


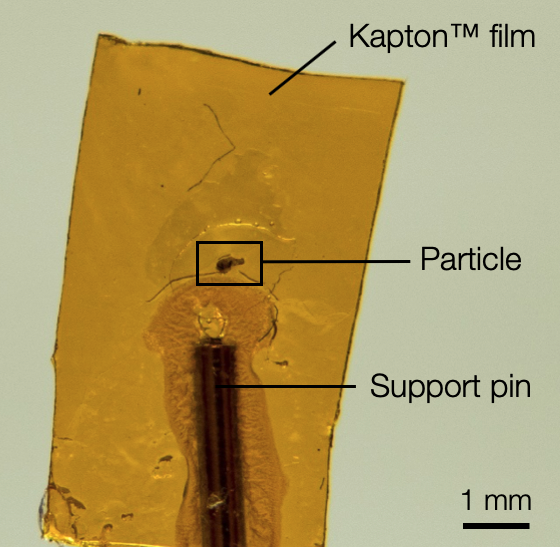


***Figure S2.*** *Image of the SR experimental setup with particle CF-01-1 enclosed within the double layered Kapton film envelope.*


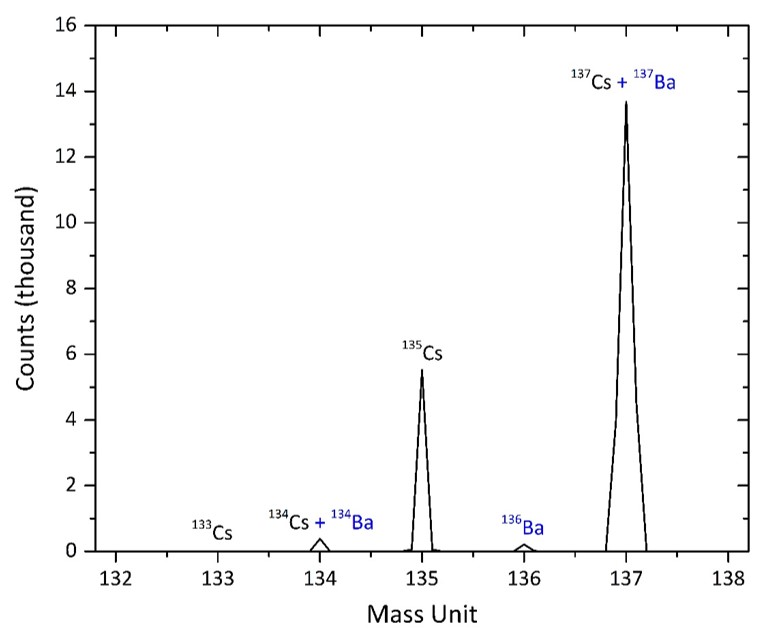


***Figure S3.*** *MS-SIMS spectrum of a 100 μm × 100 μm area of the CF-01-1 particle, across the Cs mass window.*


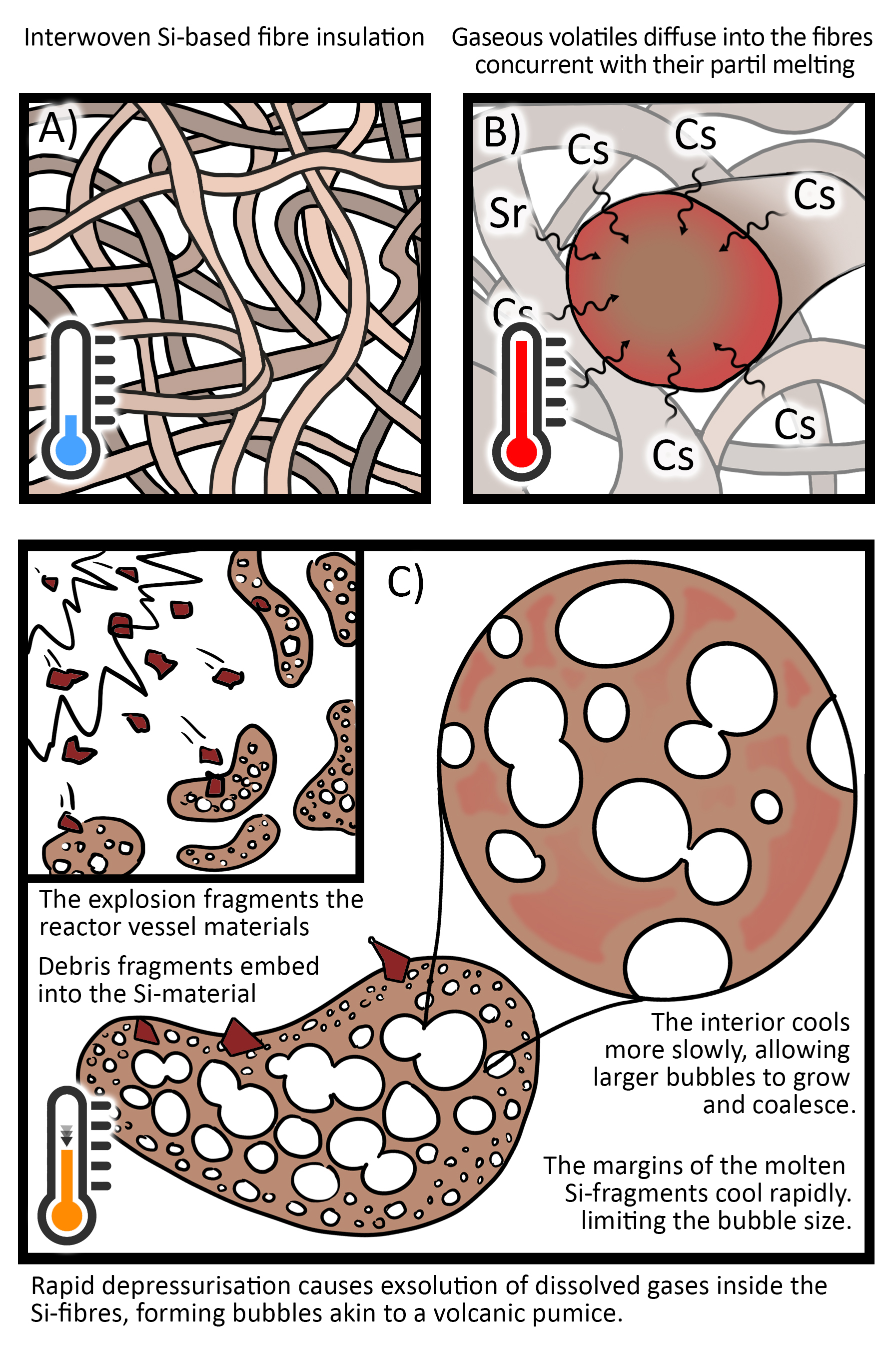


***Figure S4.*** *Schematic representation of the multi-stage formation mechanism of the radioactive particulate studied in this work.*

| **Element** | **Reference Composition Range (wt%)** | **This Particle ± 2σ (wt%)** |
| --- | --- | --- |
| C | 0.04 – 0.10 | ND |
| Mn | 0.10 – 2.00 | 0.9 ± 0.1 |
| Si | <0.75 | 2.3 ± 0.4 |
| P | <0.045 | <0.1 ± 0.1 |
| S | <0.03 | <0.1 ± 0.1 |
| Cr | 16.0 – 18.0 | 14.8 ± 1.2 |
| Mo | 2.0 – 3.0 | 2.7 ± 0.4 |
| Ni | 10.0 – 14.0 | 9.8 ± 0.9 |
| Fe | 61.0 – 72.0 | 68.0 ± 2.1 |
| Zn | 0 | 1.2 ± 0.2 |

***Table S1.*** *EDS compositional comparison (with associated uncertainties) of the protruding (angular) Fe-rich particle (within particle CF-01-1) with a reference AISI Type 316 stainless steel (MatWeb Material Property Database, 2018). It was not possible to accurately determine the carbon content of the particle inclusion through EDS analysis due to the high background of the element present within the experimental setup.*

MatWeb Material Property Database. 2018. “AISI 316H Stainless Steel Reference Properties.” http://www.matweb.com/ (Date accessed: April 24, 2018).

| **Oxide** | **Reference Composition Range (wt%)** | **This Particle ± 2σ (wt%)** |
| --- | --- | --- |
| CaO | 60.2 – 66.3 | 62.5 ± 3.3 |
| SiO_2_ | 18.2 – 23.4 | 25.1 ± 1.8 |
| Al_2_O_3_ | 2.4 – 6.3 | 2.3 ± 0.4 |
| Fe_2_O_3_ | 1.3 – 6.1 | 6.8 ± 0.5 |
| MgO | 0.6 – 4.8 | 1.4 ± 0.3 |
| Na_2_O | 0.05 – 1.2 | 0.3 ± 0.1 |
| K_2_O |  | 0.1 ± 0.1 |
| SO_3_ | 1.7 – 4.6 | 1.5 ± 0.4 |

***Table S2.*** *EDS compositional comparison (with associated uncertainties) of a Ca-rich portion of the CF-01-1 particle (identified in green in Figures 4 (a) and (b)) alongside the reference composition of a Portland-type cement (Taylor 1997).*

Taylor, HFW. 1997. *Cement Chemistry, Second Edition*. Aberdeen, Scotland: ICE Publishing.

| **Particle** | **^134^Cs (Bq) (±2σ)** | **^137^Cs (Bq) (±2σ)** | **^134^Cs/^137^Cs (±2σ)** |
| --- | --- | --- | --- |
| CF-01-R024 | 89.0 ± 2.6 | 97.0 ± 2.9 | 0.92 ± 0.03 |
| CF-01-R009 | 1,200 ± 35.0 | 1,300 ± 38.0 | 0.92 ± 0.03 |
| CF-01-T18 | 83.0 ± 2.4 | 91.0 ± 27.0 | 0.91 ± 0.02 |
| CF-01-T06 | 180.0± 5.4 | 170.0 ± 5.0 | 0.93 ± 0.03 |
| CF-01-1 | 18,000 ± 540 | 19,300 ± 580 | 0.93 ± 0.04 |

***Table S3.*** *^134^Cs and ^137^Cs activities (with associated uncertainties) for the five particles analysed during this study. All values are decay-corrected to 11^th^ March 2011.*

| **Element** | **Fibre Composition ± 2σ**  **(wt%)** |
| --- | --- |
| O | 30.2 ± 4.3 |
| Na | 11.5 ± 1.7 |
| Mg | 1.7 ± 0.3 |
| Al | 1.6 ± 0.3 |
| Si | 40.5 ± 3.8 |
| S | 0.4 ± 0.1 |
| K | 1.0 ± 0.2 |
| Ca | 11.6 ± 1.8 |
| Mn | 0.9 ± 0.3 |
| Fe | 0.7 ± 0.2 |

***Table S4.*** *EDS compositional breakdown of Rockwool fibrous insulation material (with associated uncertainties).*
